# 

**Published:** 1919-07-05

**Authors:** 


### The Book Market.

**List of New Books Received from the following publishers:—**

John Wright & Sons: "Diseases of the Ear in School Children." By James Kerr Love, M.D. 5s. 6d. net.

Longmans Green: "The Control of the Drink Trade." By Henry Carter. Cloth boards, 4s. 6d. net; cloth, limp, 2s. 6d. net.

G. Bell & Sons, Ltd.: "Fresh Hope and Health." By Cecilie Muller. 2s. net.

National League for Health, Maternity, and Child Welfare: "Mothercraft." 5s. net.

Pie Publications, Ltd.: "Printers' Pie." By Mrs. W. Hugh Spottiswoode. 1s. 6d. net.

### Medical Appointments.

THE following appointments have been made at the London Lock Hospital:—

F. A. Juler, Esq., F.R.C.S., ophthalmic surgeon; Lieut.-Colonel J. Johnston Abraham, F.R.C.S., D.S.O., honorary surgeon to out-patients; Captain David Thomson, O.B.E., M.B., Ch.B.(Edin.), D.P.H.(Camb.), research pathologist and lecturer on venereal pathology; N. S. Bonard, Esq., M.D., M.R.C.S., pathologist; Lieut.-Col. L. D. Shaw, M.B., Ch.B., house surgeon to the Male Lock Hospital; Mr. E. M. Mahon, M.R.C.S., L.R.C.P., clinical assistant to Lieut.-Col. J. J. Abraham; J. Ernest Lane, Esq., F.R.C.S., senior surgeon to the London Lock Hospital, and J. E. R. McDonagh, Esq., F.R.C.S., have, in addition to their present appointments, undertaken charge of in-patients at the Female Lock Hospital; F. T. Wild, Esq., L.D.S., dental surgeon to the London Lock Hospital.

### MATRONS' AND SISTERS' APPOINTMENTS.

The Birmingham Royal Institution for the Blind, Edgbaston.—Miss Mary E. Llewellyn has been appointed assistant matron. She was trained at Swansea General and Eye Hospital, and has been sister at Moseley Hall Hospital, Birmingham.

Tuberculosis Dispensary, Bury.—Miss Grace Mary Barker has been appointed matron. She was trained at the North Evington Infirmary, Leicester, and has been home sister at Spittals Hospital, Stoke-on-Trent.

Monsall Hospital, Manchester.—Miss S. L. Bevan has been appointed matron She was trained at the Royal Infirmary, Sheffield, and at the Leeds City Fever Hospitals; has been sister at Huddersfield Sanatorium, and sister, night sister, and assistant matron at the Monsall Hospital.

The Yarrow Convalescent Home, Broadstairs.—Miss Mabel E. Williams has been appointed matron. She was trained at the Royal Southern Hospital, Liverpool, and has since been ward sister, night sister, home sister, and assistant matron at the Leasowe Hospital for Children, Cheshire.

County Mental Hospital, Cheddleton, near Leek, Staffs.—Miss G. F. Burnell has been appointed assistant matron. She was trained at the West London Hospital, has been charge nurse at the City Hospital, Little Bromwich, Birmingham, charge nurse and acting holiday matron at the Bath Statutory Hospital, and has served in France under the Civil Hospitals Reserve.

Innes Hopkins Memorial Home, Ryton-on-Tyne.—Miss C. C. Boness has been appointed sister. She was trained at the Kingston-upon-Hull Infirmary, has been charge nurse at the Old Windsor Infirmary, and sister at the Silverdale Auxiliary Military Hospital and at the British Red Cross Hospital, Le Treport.

London Homœopathic Hospital, Great Ormond Street.—Miss E. Cummins has been appointed massage sister. She was trained at the Brownlow Hill Infirmary, Liverpool, and holds the certificates of the Central Midwives Board and of the Incorporated Society of Trained Masseuses.

Borough Hospital, Birkenhead.—Miss F. M. Williams has been appointed sister. She was trained at the Wolverhampton Infirmary, has been sister at the Cardiff Union Hospital and at the Selly Oak Infirmary, Birmingham, and has been for three and a-half years a member of Queen Alexandra's Imperial Military Nursing Service Reserve.

